# Association between life’s essential 8 and metabolic dysfunction-associated steatotic liver disease among US adults

**DOI:** 10.1186/s13690-024-01331-3

**Published:** 2024-07-05

**Authors:** Zheng Wang, Bohan Huang, Yixuan Ding, Feng Cao, Fei Li, Prof Fei Li

**Affiliations:** Department of General Surgery, Xuanwu Hospital, Capital Medical University, No.45 Changchun Street, Xicheng District, Beijing, 100053 China

**Keywords:** NHANES, Cardiovascular health, Public health, Physical activity, MASLD

## Abstract

**Background:**

Metabolic dysfunction-associated steatotic liver disease(MASLD) is the most common cause of chronic liver disease. Clinical evidences have demonstrated the link between MASLD and the increased risk of cardiovascular disease (CVD) development. We aimed to investigate the relationship between Life’s Essential 8 (LE8), an enhanced approach to assessing cardiovascular health(CVH), and MASLD.

**Methods:**

Data were extracted from the National Health and Nutrition Examination Survey (NHANES) in 2017–2020 cycles. MASLD was assessed by the latest diagnostic criteria. LE8 scores (range 0–100) were obtained from measurements based on American Heart Association definitions, divided into health factor and health behavior scores. Multivariable logistic and restricted cubic spline models were used to assess the associations.

**Results:**

5646 participants were included based on the inclusion and exclusion criteria, 2616 (46.33%) participants were diagnosed with MASLD. After adjusting for confounding variables, higher LE8 scores were associated with a lower risk of MASLD (OR = 0.19, 95%CI 0.17–0.21; *P* < 0.001), similar associations were also observed between health behavior and health factor scores with MASLD. Subgroup analyses illustrated that the negative association between LE8 score and MASLD was stronger among younger, non − Hispanic White, and never married participants.

**Conclusions:**

In this nationally representative sample of U.S. adults, LE8 scores, health behavior scores, and health factor scores were negatively associated with the prevalence of MASLD in non-linear fashions. Subjects maintaining ideal health factors and health behaviors are less likely to develop MASLD. Public health policies are needed to advocate healthy behaviors and factors.

**Supplementary Information:**

The online version contains supplementary material available at 10.1186/s13690-024-01331-3.

**Table:** 

**Text box 1. Contributions to the literature**
• Metabolic dysfunction-associated steatotic liver disease(MASLD) is the most common cause of chronic liver disease worldwide. The burden of MASLD and its complications is projected to continue to increase in the coming years.
• This study investigated the correlation between Life’s Essential 8 (LE8) score and the prevalence of MASLD among US adults.
• Public health policies are needed to appeal to public maintaining ideal health behaviors and health factors with the findings of this study.

## Background

Metabolic dysfunction-associated steatotic liver disease(MASLD), formerly known as non-alcoholic fatty liver disease (NAFLD), is the latest term for steatotic liver disease associated with metabolic syndrome [1]. MASLD is the most common cause of chronic liver disease and is the leading cause of liver-related morbidity and mortality, with a global prevalence increasing from 22 to 37% from 1991 to 2019 [2, 3]. The burden of MASLD and its complications, including hepatocellular carcinoma, is projected to continue to increase in the coming years.

MASLD, by definition, is associated with the metabolic syndrome—specifically, obesity, dyslipidemia, hypertension, and insulin resistance, all substantial risk factors for CVD [4]. Clinical evidences have demonstrated the link between MASLD and the increased risk of CVD development, including hypertension, atherosclerosis, cardiomyopathies, and chronic kidney disease [5–7]. In fact, cardiovascular events and complications of CVD stands as the primary cause of mortality in MASLD patients, surpassing the liver disease itself [8, 9]. CVD risk reduction is vital for the prevention and management of MASLD.

The American Heart Association has recently introduced an enhanced approach to assessing CVH: LE8 [10]. This scoring system encompasses components that partially overlap with risk factors for MASLD, including blood pressure, lipid profile, glucose level, physical activity, and diet [11]. Previous studies have suggested that adherence to optimal LE8 levels may be beneficial to reduce the burden of NAFLD as well as CVD [12–14]. Indeed, the diagnostic criteria for MASLD have undergone significant changes compared to those for NAFLD, whether the association between the LE8 score and MASLD still exists is unknown. Therefore, this study aimed to evaluate the association between LE8 score and the prevalence of MASLD using the NHANES data.

## Methods

### Study design and participants

The NHANES is a national cross-sectional study in the United States. It can be accessed through the Centers for Disease Control and Prevention National Center for Health Statistics (NCHS; https://www.cdc.gov/nchs/). The NHANES employs a complex, multistage probability sample design and conducts surveys of 5000 people every two years on average. Data from 2017 to 2020 were combined to perform this cross-sectional analysis. This study followed the Strengthening the Reporting of Observational Studies in Epidemiology (STROBE) reporting guideline [15].

**Fig. 1:**
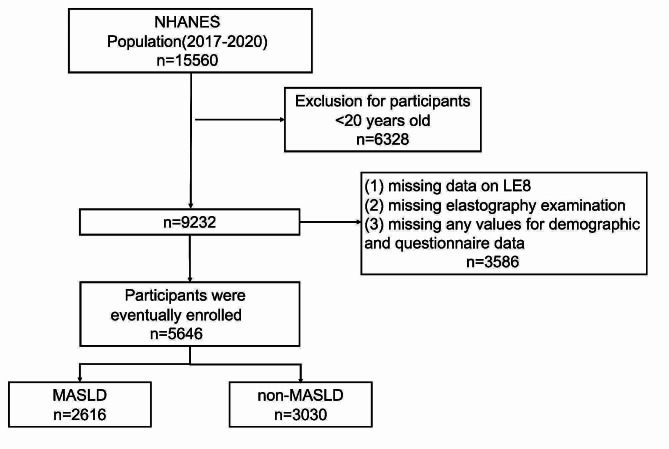
Flowchart of study participants

This cross-sectional study included 15,560 participants from the nationally representative consecutive NHANES 2017–2020, individuals were excluded if (1) under 20 years of age; (2) missing data on LE8; (3) missing elastography examination; (4) missing any values for demographic and questionnaire data. Ultimately, a total of 5646 participants were finally enrolled. Figure 1 shows the data processing details.

### Study covariates

Covariates consisted of demographic characteristics, several factors previously displayed or assumed to be associated with LE8 or MASLD, including gender, age, race, education, marital status, poverty-to-income ratio (PIR), body mass index (BMI), low-density lipoprotein cholesterol (LDL), high-density lipoprotein cholesterol (HDL), plasma triglyceride, waist circumference, fasting serum glucose, glycohemoglobin type A1c (HbA1c), blood pressure, drug treatment, alcohol use, diabetes mellitus, cigarette use.

### Definition of MASLD

Hepatic steatosis in this study was defined by the controlled attenuation parameter (CAP), obtained via vibration controlled transient elastography (VCTE), which is a validated tool for measuring steatosis in participants with fatty liver [16]. CAP ≥ 268 dB/m was defined as significant hepatic steatosis [17]. The diagnosis of MASLD is based on hepatic steatosis and requires the fulfillment of at least one of five: (1) BMI ≥ 25 kg/m^2^ (≥ 23 kg/m^2^ in Asian) or waist circumference > 94 cm in men, > 80 cm in women, or ethnicity adjusted; (2) Fasting serum glucose ≥ 100 mg/dL (≥ 5.6 mmol/L) or 2-hour post-load glucose level ≥ 140 mg/dL(≥ 7.8 mmol/L) or HbA1c ≥ 5.7% or on specific drug treatment; (3) Blood pressure ≥ 130/85 mmHg or specific drug treatment; (4) Plasma triglycerides ≥ 150 mg/dL (≥ 1.70 mmol/L) or specific drug treatment; (5) Plasma HDL cholesterol < 40 mg/dL (< 1.0 mmol/L) for men and < 50 mg/dL (< 1.3 mmol/L) for women or specific drug treatment [1].

### Measurement of LE8

The components of LE8 include 4 health behaviors (diet, physical activity, nicotine exposure, and sleep duration) and 4 health factors (body mass index, non-high-density-lipoprotein cholesterol, blood glucose, and blood pressure). Each metric has a scoring algorithm ranging from 0 to 100 points, allowing generation of a new composite CVH score (the unweighted average of all components) that also varies from 0 to 100 points. Detailed algorithms for calculating the LE8 scores for each of the metrics to NHANES data have been previously published and can be found in Supplementary File 1. The overall CVH scores of 80 to 100 be considered high CVH; 50 to 79, moderate CVH; and 0 to 49 points, low CVH [10].

Dietary indicators were assessed using the Healthy Eating Index (HEI) 2015 measured by the subjects’ 24-h dietary review [18]. Physical activity, nicotine exposure and sleep data were collected through a self-report questionnaire. Details of the questionnaire can be found in Supplemental File 2. The body measures data include height and weight, height was defined as standing height. Those data were collected, in the Mobile Examination Center (MEC), by trained health technicians. The health technician was assisted by a recorder during the body measures examination. BMI was obtained by dividing weight (kilograms) by the square of height (meters). All blood pressure determinations (systolic and diastolic) are taken in the MEC. After resting quietly in a seated position for 5 min and after the participant’s maximum inflation level (MIL) has been determined, three consecutive blood pressure readings are obtained. NHANES collected the fasting blood sample for laboratory analysis, including plasma glucose, HbA1c, HDL, and total cholesterol. Non-HDL cholesterol was obtained by subtracting HDL from total plasma cholesterol.

### Statistical analysis

Due to the complexity of the NHANES sampling design, appropriate weights were used for the sample analysis. For baseline characterization, weighted means (standard errors) were used for continuous variables, and sample sizes (weighted percentages) were used for categorical variables. Survey-weighted multivariable logistic regressions were used to investigate the independent association of LE8 and different degrees of CVH with the risks of MASLD. Crude models did not adjust for any potential confounders. Model 1 adjusted for age, sex, race. Model 2 was further adjusted for education, marital status. Restricted cubic spline (RCS) was also used to further validate the link between LE8 scores and its subscales score with MASLD. To examine different subpopulations at baseline, subgroup analyses were performed by gender, age strata, race, education levels, marital status and ratio of family income to poverty. For sensitivity analyses, we excluded the individuals with comorbidity (including CVD, COPD, and cancer) to assess the robustness of our findings. Statistical significance was ascertained by a two-sided *P* value of < 0.05. All statistical analyses were performed using R software, version 4.2.2 (R Core Team, Vienna, Austria).

## Results

### Participant characteristics

In this study, 5646 participants were included based on the inclusion and exclusion criteria, and the average age of the participants was 50.2 ± 1.7 years. Among these participants, 49.45% were male, 50.55% were female, 11.07% were Mexican American, 37.90% were non-Hispanic white, 24.76% were non-Hispanic black, 9.85% were other Hispanic, and 16.42% were from other races. 2616 (46.33%) participants were diagnosed with MASLD.

For demographic sociology, participants with MASLD were older, more male, more white, less educated, lower education levels, and were married/living with partner. Regarding LE8 scores, participants with MASLD have a lower score, as well as in the health behaviors score, and health factors score, while the sleep duration scores had no significant difference among the two groups.

Baseline characteristics of the study population were summarized by the category of MASLD status in Table 1.

**Table 1:** Baseline characteristics

	Overall (*N* = 5646)		MASLD (*N* = 2616)	non-MASLD (*N* = 3030)	*P* value
Age, years, (SE)		50.2 (1.7)	53.1 (1.6)	48.2 (1.8)	< 0.001
Gender, n (%)					< 0.001
male		2792 (49.45)	1429 (54.63)	1363 (44.98)	
female		2854 (50.55)	1187 (45.37)	1667 (55.02)	
Race, n (%)					< 0.001
Mexican American		625 (11.07)	389 (14.87)	236 (7.79)	
Non-Hispanic White		2140 (37.90)	1049 (40.10)	1091 (36.01)	
Non-Hispanic Black		1398 (24.76)	541 (20.68)	857 (28.28)	
Other Hispanic		556 (9.85)	265 (10.13)	291 (9.60)	
Other Race		927 (16.42)	372 (14.22)	555 (18.32)	
Education, n (%)					< 0.001
High school or less		2257 (39.98)	1102 (42.13)	1155 (38.12)	
Some college or associates degree		1943 (34.41)	933 (35.67)	1010 (33.33)	
College graduate or above		1446 (25.61)	581 (22.21)	865 (28.55)	
Marital status, n (%)					< 0.001
Married/Living with Partner		3326 (58.91)	1644 (62.84)	1682 (55.51)	
Widowed/Divorced/Separated		1249 (22.12)	586 (22.40)	663 (21.88)	
Never married		1071 (18.97)	386 (14.76)	685 (22.61)	
Ratio of family income to poverty		2.65 ± 1.63	2.66 ± 1.61	2.65 ± 1.65	0.869
LE8 score		71.0 (1.3)	64.3 (1.1)	76.2 (1.2)	< 0.001
Health factors score		69.5 (1.9)	60.2 (1.6)	77.5 (1.7)	< 0.001
Health behavior score		65.3 (1.1)	63.2 (1.1)	67.8 (1.1)	< 0.001
Blood pressure score		66.9 (3.4)	61.0 (3.4)	71.1 (3.4)	< 0.001
Blood glucose score		79.7 (2.8)	71.9 (3.0)	87.0 (2.3)	< 0.001
Blood lipids score		73.6 (2.9)	69.8 (3.0)	77.2 (2.8)	< 0.001
Body mass index score		57.4 (3.4)	38.5 (2.9)	72.4 (3.0)	< 0.001
Sleep health score		84.3 (2.4)	83.7 (2.4)	84.6 (2.4)	0.768
Nicotine exposure score		60.2 (2.9)	61.3 (2.8)	59.5 (3.0)	0.034
Physical activity score		75.7 (1.5)	67.7 (1.6)	81.2 (1.1)	< 0.001
HEI-2015 diet score		43.2(2.3)	41.1 (2.6)	45.6 (3.1)	< 0.001

MASLD: Metabolic dysfunction-associated steatotic liver disease; LE8: life’s essential 8; HEI: healthy eating index

Data were presented as weighted percentages or means (95% confidence intervals)

### LE8 score and MASLD

**Fig. 2:**
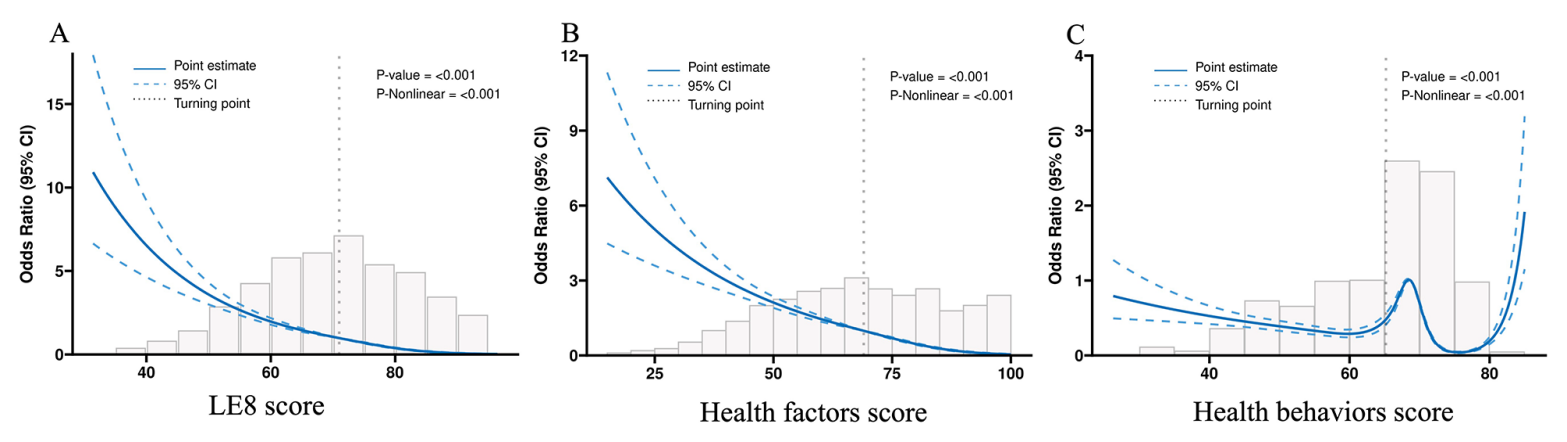
Analysis of restricted cubic spline regression. **A** LE8 score. **B** Health factors score. **C** Health behaviors score

Table 2 showed the results of the multivariable regression analysis between LE8 score and MASLD. After adjusting for confounding variables, the association was significant (OR = 0.19, 95%CI 0.17–0.21; *P* < 0.001) between LE8 score and MASLD. Compared with the low CVH group, the OR of MASLD were 0.21 (95% CI 0.15–0.29; *P* < 0.001) in the moderate CVH group and 0.03 (95%CI 0.02–0.04; *P* < 0.001) in the high CVH group, respectively. The RCS analysis suggested a non-linear association of LE8 with MASLD (Fig. 2A).

**Table 2:** Association of the LE8 scores with MASLD

	Crude model		Model 1		Model 2	
	OR(95% CI)	*P* value	OR(95% CI)	*P* value	OR(95% CI)	*P* value
LE8 score	0.20 (0.18–0.22)	< 0.001	0.19 (0.17–0.22)	< 0.001	0.19 (0.17–0.21)	< 0.001
Low LE8(0–49)	Reference		Reference		Reference	
Moderate LE8(50–79)	0.26 (0.19–0.36)	< 0.001	0.22 (0.16–0.31)	< 0.001	0.21 (0.15–0.29)	< 0.001
High LE8(80–100)	0.04 (0.03–0.05)	< 0.001	0.03 (0.02–0.04)	< 0.001	0.03 (0.02–0.04)	< 0.001

Crude model: unadjusted model;

Model 1: Adjusted for age, sex, race;

Model 2: Adjusted for age, sex, race, marital status, education

LE8, life’s essential 8; MASLD, Metabolic dysfunction-associated steatotic liver disease

### Relationship of health factors /health behaviors with MASLD

Table 3 presented the results of the multivariable regression analysis of health factor scores and health behavior scores with MASLD. After adjusting for confounding variables, this association was significant (OR = 0.94, 95%CI 0.93–0.94; *P* < 0.001) between health factor scores and MASLD. Compared with the low health factor scores group, the OR of MASLD were 0.38 (95% CI 0.32–0.45; *P* < 0.001) in the moderate health factor scores group and 0.06 (95%CI 0.05–0.07; *P* < 0.001) in the high health factor scores group, respectively. The RCS analysis suggested a non-linear association of health factor scores with MASLD (Fig. 2B).

**Table 3:** Relationship between health behaviors score/health factors score and MASLD

	Crude model		Model 1		Model 2	
	OR(95% CI)	*P* value	OR(95% CI)	*P* value	OR(95% CI)	*P* value
Health factors score	0.94 (0.94–0.95)	< 0.001	0.94 (0.93–0.94)	< 0.001	0.94 (0.93–0.94)	< 0.001
Classification						
Low(0–49)	Reference		Reference		Reference	
Moderate(50–79)	0.42 (0.36–0.49)	< 0.001	0.38 (0.32–0.45)	< 0.001	0.38 (0.32–0.45)	< 0.001
High(80–100)	0.07 (0.05–0.08)	< 0.001	0.06 (0.05–0.07)	< 0.001	0.06 (0.05–0.07)	< 0.001
Health behaviors score	0.96 (0.96–0.97)	< 0.001	0.96 (0.95–0.96)	< 0.001	0.96 (0.95–0.96)	< 0.001
Classification						
Low(0–49)	Reference		Reference		Reference	
Moderate(50–79)	0.47 (0.40–0.56)	< 0.001	0.44 (0.37–0.52)	< 0.001	0.43 (0.36–0.51)	< 0.001
High(80–100)	0.03 (0.01–0.05)	< 0.001	0.02 (0.01–0.04)	< 0.001	0.02 (0.01–0.04)	< 0.001

Crude model: unadjusted model;

Model 1: Adjusted for age, sex, race;

Model 2: Adjusted for age, sex, race, marital status, education

LE8, life’s essential 8; MASLD, Metabolic dysfunction-associated steatotic liver disease

After multivariable adjustment, the association between health behavior scores and MASLD was significant (OR = 0.96, 95%CI 0.95–0.96; *P* < 0.001). Compared with the low health behavior scores group, the OR of MASLD were 0.43 (95% CI 0.36–0.51; *P* < 0.001) in the moderate health behavior scores group and 0.02 (95%CI 0.01–0.04; *P* < 0.001) in the high health behavior scores group, respectively. The RCS analysis suggested a non-linear association of health behavior scores with MASLD (Fig. 2C).

### Subgroup analyses of LE8 with MASLD

**Fig. 3:**
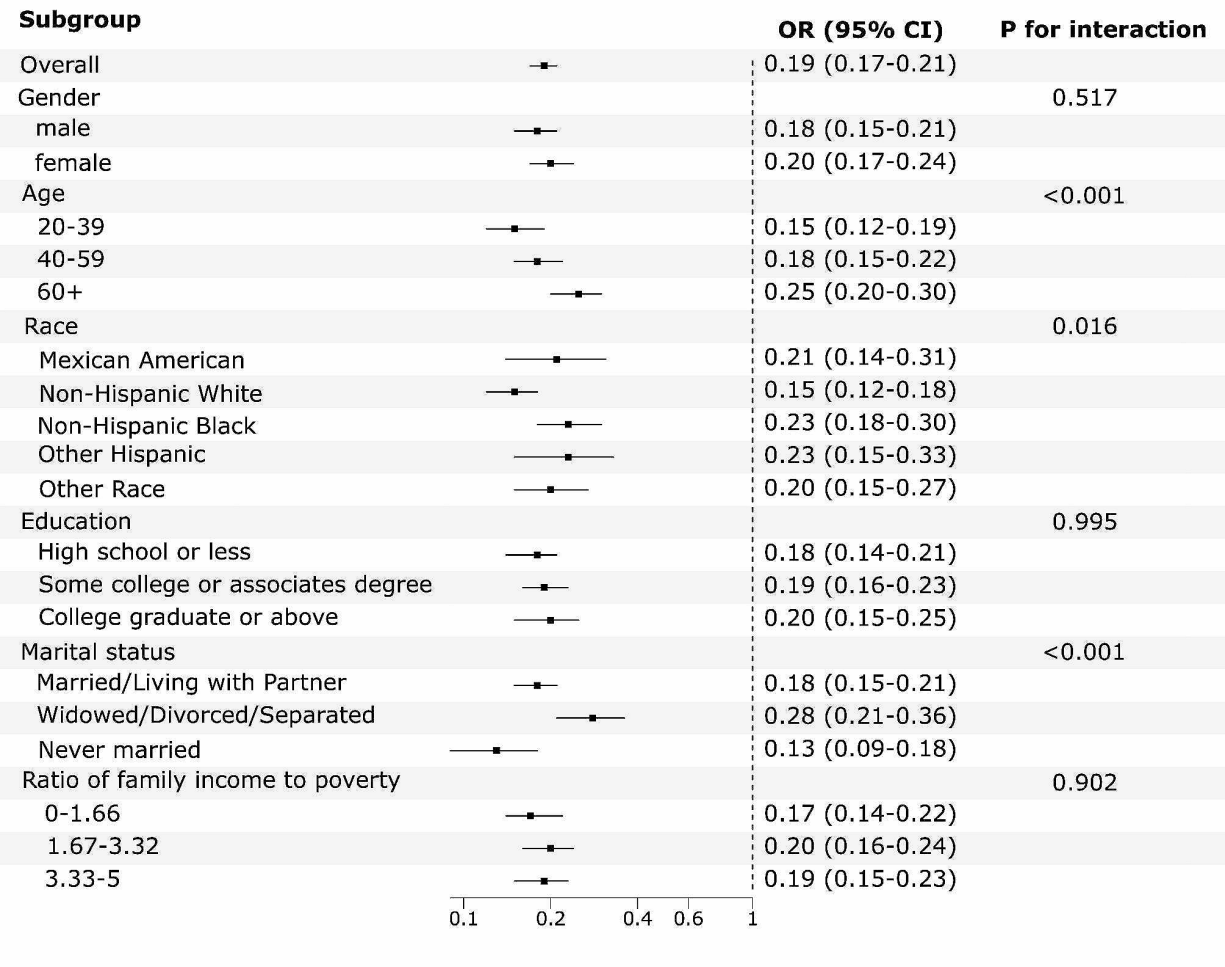
Subgroup analysis of the association of the LE8 scores and the presence of MASLD

Further subgroup analysis revealed that the LE8 score was negatively associated with MASLD in all subgroups, as shown in Fig. 3. LE8 score was shown to correlate significantly with MASLD in subgroups stratified by age, race, and marital status (*P* < 0.05). The negative association between LE8 score and MASLD was more pronounced in younger participants(OR = 0.15, 95%CI 0.12–0.19), Non-Hispanic White individuals, (OR = 0.15, 95%CI 0.12–0.18) and never married participants(OR = 0.13, 95%CI 0.09–0.18).

## Discussion

To elucidate the relationship between LE8 scores and MASLD, we carried out a cross-sectional analysis of 5646 participants from the NHANES cohort. Negative associations were revealed between the LE8 score and its health factors and health behavior subscales with MASLD. The prevalence of MASLD were progressively decreased with an increased IL8 score. Subgroup analyses illustrated that the negative association between LE8 score and MASLD was stronger among younger, non − Hispanic White, and never married participants. The association remained significant after adjusting confounding variables.

As mentioned above, CVD is the leading cause of death in patients with MASLD. A prospective study on a cohort of patients with MASLD found the rate of cardiovascular event to be 2.03 per 100 person-years while the rate of liver-related event was 0.43 per 100 person-years [19]. Various studies have evaluated the relationship between LE8 and NAFLD [20, 21]. However, the most significant difference between NAFLD and the diagnosis of MASLD is not the formal recognition of metabolic dysregulatory pathways in the development of the disease, but rather the removal of exclusion of concurrent liver disease to entertain the diagnosis [22, 23]. Unlike NAFLD, MASLD defines the disease positively, unrelated to the presence or absence of other liver diseases, and is closely associated with metabolic syndrome [24]. This distinction emphasizes that the definition of MASLD may better capture the health behaviors and characteristics of the body compared to the definition of NAFLD [25].

This study represents the inaugural exploration of the relationship between LE8 and MASLD. Utilizing the latest diagnostic criteria for MASLD, our findings support the association between LE8 and MASLD. MASLD’s etiology is intricate, involving interactions among diverse factors, including lifestyle, dietary elements, and individual inheritance [26]. We observed that a higher LE8 score correlated with a lower prevalence of MASLD in U.S. adults, with both health factors and health behaviors scores exhibiting a similar trend. These results underscore the potential effectiveness of LE8 in promoting hepatic health. While the precise mechanisms linking LE8 and MASLD remain unidentified, MASLD, characterized by obesity, insulin resistance, and hepatic steatosis, impacts cardiovascular health through various pathways, including its association with obesity and diabetes, heightened inflammation, oxidative stress, and alterations in hepatic metabolites and hepatokines [27]. Symptoms of MASLD can be difficult to recognize, screening methods are being investigated by which to efficiently and noninvasively identify cases. For patients with low LE8 score, whether it is necessary to perform liver biopsy and ultrasound elastography need more research to analyze the relationship between MASLD and CVD.

Life intervention is the cornerstone in the management of MASLD [28]. Dietary habits, including caloric intake and specific nutrients, significantly contribute to the development of MASLD. Epidemiological studies, clinical trials, and animal studies indicate that excess dietary carbohydrates, particularly fructose, are risk factors for hepatic steatosis [29]. Calorie restriction and increased physical activity, leading to weight loss and an increase in muscle mass, can significantly improve MASLD [30, 31]. The increase in muscle mass also correlates with improved blood pressure, glycemic control, lipid profiles, and a reduced risk of cardiovascular disease [32–34]. No pharmacological agent has been approved for the treatment of MASLD. But its association with increased risks of type 2 diabetes, cardiovascular disease, dyslipidemia, and sleep disturbances underscores the importance of managing these comorbidities effectively [35]. In addition, the results of this study further demonstrate that adopting healthy behaviors, including proper diet, physical activity, abstaining from smoking, and ensuring adequate sleep, can act as preventive measures against MASLD. LE8 emerges as an effective means to monitor public and individual health and a strong indicator of the extraordinary potential of primordial prevention strategies to improve and extend countless lives [10].

The current study has some limitations. This study was conducted via a survey, and therefore, the answers could be influenced by self-reporting bias. Hepatic steatosis in this study was defined by the CAP, obtained via VCTE, which is a validated tool for measuring steatosis in participants with fatty liver, and although several studies have demonstrated the extremely high accuracy, it still cannot be the same as biopsy. Dietary indicators were assessed using the HEI 2015 measured by the subjects’ 24-h dietary review,, which are susceptible to recall bias. Some advantages of the study must be stated. We utilized the national representative large sample which allows us to perform subgroup analyses between LE8 and MASLD in different populations. MASLD was defined by the latest diagnostic criteria, making the participants more comprehensive. As a result, extrapolation of the results to a broader population has a high degree of reliability.

## Conclusion

In this nationally representative sample of U.S. adults, LE8 scores, health behavior scores, and health factor scores were negatively associated with the prevalence of MASLD in non-linear fashions. Subjects maintaining ideal health factors and health behaviors are less likely to develop MASLD. Public health policies are needed to advocate healthy behaviors and factors.

### Electronic supplementary material

Below is the link to the electronic supplementary material.

Supplementary Material 1

Supplementary Material 2

## Data Availability

The dataset supporting the conclusions of this article is available for people to collect data from the official website for users and researchers worldwide (https://www.cdc.gov/nchs/).
