# Supplementary material for: Association between life’s essential 8 and metabolic dysfunction-associated steatotic liver disease among US adults
Source: Arch Public Health. 2024 Jul 5;82:101. doi: 10.1186/s13690-024-01331-3 (PMC11225315; doi:10.1186/s13690-024-01331-3)
Supplement: Supplementary file 2 — Supplementary Material 2 [file 13690_2024_1331_MOESM2_ESM.pdf]

PHYSICAL ACTIVITY AND PHYSICAL FITNESS – PAQ
Target Group: SPs 2+

BOX 1

CHECK ITEM PAQ.700:
IF SP AGE 2-11 OR 16-17, GO TO PAQ706.
IF SP AGE <2 OR SP 12-15, GO TO NEXT SECTION.
IF SP AGE 18+, CONTINUE.

PAQ.605 Next I am going to ask you about the time {you spend/SP spends} doing different types of physical activity in a typical week.

Think first about the time {you spend/he spends/she spends} doing work. Think of work as the things that {you have/he has/she has} to do such as paid or unpaid work, household chores, and yard work.

Does {your/SP's} work involve **vigorous**-intensity activity that causes **large increases** in breathing or heart rate like carrying or lifting heavy loads, digging or construction work for **at least 10 minutes continuously**?

YES	1	
NO	2	(PAQ.620)
REFUSED	7	(PAQ.620)
DON'T KNOW	9	(PAQ.620)

PAQ.610 In a typical week, on how many days {do you/does SP} do **vigorous**-intensity activities as part of {your/his/her} work?

PROBE IF NEEDED: Vigorous-intensity activity causes large increases in breathing or heart rate and is done for at **least 10 minutes continuously**.

INTERVIEWER: REMEMBER, WE ARE ONLY ASKING ABOUT WORK AND CHORES IN THIS QUESTION.

HARD EDIT: 1-7.
ERROR MESSAGE: THE NUMBER OF DAYS SHOULD BE BETWEEN 1 AND 7.

|___|___|
ENTER NUMBER OF DAYS

REFUSED	77	(PAQ.620)
DON'T KNOW	99	(PAQ.620)

PAQ.615 Q/U How much time {do you/does SP} spend doing **vigorous**-intensity activities at work on a typical day?

PROBE IF NEEDED: Think about a typical day when {you do/he does/she does} vigorous-intensity activities during {your/his/her} work.

PROBE IF NEEDED: Vigorous-intensity activity causes large increases in breathing or heart rate and is done for at **least 10 minutes continuously**.

INTERVIEWER: REMEMBER, WE ARE ONLY ASKING ABOUT WORK AND CHORES.

SOFT EDIT: >4 HOURS.
ERROR MESSAGE: INTERVIEWER, YOU HAVE RECORDED THAT THE SP SPENDS MORE THAN 4 HOURS DOING VIGOROUS-INTENSITY ACTIVITIES AT WORK ON A TYPICAL DAY. PLEASE CONFIRM WITH SP THAT OVER 4 HOURS IS CORRECT.

HARD EDIT: ≥24 HOURS.
HARD EDIT: <10 MINUTES.
ERROR MESSAGE: THE TIME SHOULD BE 10 MINUTES OR MORE, BUT LESS THAN 24 HOURS.

|___|___|___|
ENTER NUMBER OF MINUTES OR HOURS

REFUSED	7777	(PAQ.620)
DON'T KNOW	9999	(PAQ.620)

ENTER UNIT

MINUTES	1
HOURS	2

PAQ.620 Does {your/SP's} work involve **moderate**-intensity activity that causes **small increases** in breathing or heart rate such as brisk walking or carrying light loads for **at least 10 minutes continuously**?

YES	1	
NO	2	(PAQ.635)
REFUSED	7	(PAQ.635)
DON'T KNOW	9	(PAQ.635)

PAQ.625 In a typical week, on how many days {do you/does SP} do **moderate**-intensity activities as part of {your/his/her} work?

PROBE IF NEEDED: Moderate-intensity activity causes small increases in breathing or heart rate and is done for at **least 10 minutes continuously**.

INTERVIEWER: REMEMBER, WE ARE ONLY ASKING ABOUT WORK AND CHORES.

HARD EDIT: 1-7.
ERROR MESSAGE: THE NUMBER OF DAYS SHOULD BE BETWEEN 1 AND 7.

|___|___|
ENTER NUMBER OF DAYS

REFUSED 77 (PAQ.635)
DON'T KNOW 99 (PAQ.635)

PAQ.630
Q/U
How much time {do you/does SP} spend doing **moderate**-intensity activities at work on a typical day?

PROBE IF NEEDED: Think about a typical day when {you do/he does/she does} moderate-intensity activities during {your/his/her} work.

PROBE IF NEEDED: Moderate-intensity activity causes small increases in breathing or heart rate and is done for at **least 10 minutes continuously**.

INTERVIEWER: REMEMBER, WE ARE ONLY ASKING ABOUT WORK AND CHORES.

SOFT EDIT: >4 HOURS.
ERROR MESSAGE: INTERVIEWER, YOU HAVE RECORDED THAT THE SP SPENDS MORE THAN 4 HOURS DOING MODERATE-INTENSITY ACTIVITIES AT WORK ON A TYPICAL DAY. PLEASE CONFIRM WITH SP THAT OVER 4 HOURS IS CORRECT.

HARD EDIT: ≥24 HOURS.
HARD EDIT: <10 MINUTES.
ERROR MESSAGE: THE TIME SHOULD BE 10 MINUTES OR MORE, BUT LESS THAN 24 HOURS.

|___|___|___|
ENTER NUMBER OF MINUTES OR HOURS

REFUSED 7777 (PAQ.635)
DON'T KNOW 9999 (PAQ.635)

|___|
ENTER UNIT

MINUTES 1
HOURS 2

PAQ.635 The next questions exclude the physical activities at work that you have already mentioned. Now I would like to ask you about the usual way {you travel/SP travels} to and from places. For example to work, for shopping, to school.

In a typical week {do you/does SP} walk or use a bicycle for **at least 10 minutes continuously** to get to and from places?

YES	1	
NO	2	(PAQ.650)
REFUSED	7	(PAQ.650)
DON'T KNOW	9	(PAQ.650)

PAQ.640 In a typical week, on how many days {do you/does SP} walk or bicycle for **at least 10 minutes continuously** to get to and from places?

HARD EDIT: 1-7.
ERROR MESSAGE: THE NUMBER OF DAYS SHOULD BE BETWEEN 1 AND 7.

|___|___|
ENTER NUMBER OF DAYS

REFUSED	77(PAQ.650)
DON'T KNOW	99(PAQ.650)

PAQ.645 Q/U How much time {do you/does SP} spend walking or bicycling for travel on a typical day?

PROBE IF NEEDED: Think about a typical day when {you walk or bicycle/SP walks or bicycles} for travel.

SOFT EDIT: >4 HOURS.
ERROR MESSAGE: INTERVIEWER, YOU HAVE RECORDED THAT THE SP SPENDS MORE THAN 4 HOURS WALKING OR BICYCLING TO GET TO AND FROM PLACES ON A TYPICAL DAY. PLEASE CONFIRM WITH SP THAT OVER 4 HOURS IS CORRECT.

HARD EDIT: ≥24 HOURS.
HARD EDIT: <10 MINUTES.
ERROR MESSAGE: THE TIME SHOULD BE 10 MINUTES OR MORE, BUT LESS THAN 24 HOURS.

|___|___|___|
ENTER NUMBER OF MINUTES OR HOURS

REFUSED	7777	(PAQ.650)
DON'T KNOW	9999	(PAQ.650)

|___|
ENTER UNIT

MINUTES	1
HOURS	2

PAQ.650 The next questions exclude the work and transportation activities that you have already mentioned. Now I would like to ask you about sports, fitness and recreational activities.

In a typical week {do you/does SP} do any **vigorous**-intensity sports, fitness, or recreational activities that cause **large increases** in breathing or heart rate like running or basketball for **at least 10 minutes continuously**?

YES	1	
NO	2	(PAQ.665)
REFUSED	7	(PAQ.665)
DON'T KNOW	9	(PAQ.665)

PAQ.655 In a typical week, on how many days {do you/does SP} do **vigorous**-intensity sports, fitness or recreational activities?

PROBE IF NEEDED: Vigorous-intensity activity causes large increases in breathing or heart rate and is done for at **least 10 minutes continuously**.

HARD EDIT: 1-7.
ERROR MESSAGE: THE NUMBER OF DAYS SHOULD BE BETWEEN 1 AND 7.

|___|___|
ENTER NUMBER OF DAYS

REFUSED	77	(PAQ.665)
DON'T KNOW	99	(PAQ.665)

PAQ.660 Q/U How much time {do you/does SP} spend doing **vigorous**–intensity sports, fitness or recreational activities on a typical day?

PROBE IF NEEDED: Think about a typical day when {you do/SP does} vigorous-intensity sports, fitness or recreational activities.

SOFT EDIT: >4 HOURS.
ERROR MESSAGE: INTERVIEWER, YOU HAVE RECORDED THAT THE SP SPENDS MORE THAN 4 HOURS DOING VIGOROUS-INTENSITY RECREATIONAL ACTIVITIES ON A TYPICAL DAY. PLEASE CONFIRM WITH SP THAT OVER 4 HOURS IS CORRECT.

HARD EDIT: ≥24 HOURS.
HARD EDIT: <10 MINUTES.
ERROR MESSAGE: THE TIME SHOULD BE 10 MINUTES OR MORE, BUT LESS THAN 24 HOURS.

|___|___|___|
ENTER NUMBER OF MINUTES OR HOURS

REFUSED	7777	(PAQ.665)
DON'T KNOW	9999	(PAQ.665)

|___|
ENTER UNIT

MINUTES	1
HOURS	2

PAQ.665 In a typical week {do you/does SP} do any **moderate**-intensity sports, fitness, or recreational activities that cause a **small increase** in breathing or heart rate such as brisk walking, bicycling, swimming, or golf for **at least 10 minutes continuously**?

YES	1	
NO	2	(PAQ.680)
REFUSED	7	(PAQ.680)
DON'T KNOW	9	(PAQ.680)

PAQ.670 In a typical week, on how many days {do you/does SP} do **moderate**-intensity sports, fitness or recreational activities?

PROBE IF NEEDED: Moderate-intensity sports, fitness or recreational activities cause small increases in breathing or heart rate and is done for at **least 10 minutes continuously**.

HARD EDIT: 1-7.
ERROR MESSAGE: THE NUMBER OF DAYS SHOULD BE BETWEEN 1 AND 7.

|___|___|
ENTER NUMBER OF DAYS

REFUSED	77	(PAQ.680)
DON'T KNOW	99	(PAQ.680)

PAQ.675 Q/U How much time {do you/does SP} spend doing **moderate**-intensity sports, fitness or recreational activities on a typical day?

PROBE IF NEEDED: Think about a typical day when {you do/SP does} moderate-intensity sports, fitness or recreational activities.

PROBE IF NEEDED: Moderate-intensity sports, fitness or recreational activities cause small increases in breathing or heart rate and is done for at **least 10 minutes continuously**.

SOFT EDIT: >4 HOURS.
ERROR MESSAGE: INTERVIEWER, YOU HAVE RECORDED THAT THE SP SPENDS MORE THAN 4 HOURS DOING MODERATE-INTENSITY RECREATIONAL ACTIVITIES ON A TYPICAL DAY. PLEASE CONFIRM WITH SP THAT OVER 4 HOURS IS CORRECT.

HARD EDIT: ≥24 HOURS.
HARD EDIT: <10 MINUTES.
ERROR MESSAGE: THE TIME SHOULD BE 10 MINUTES OR MORE, BUT LESS THAN 24 HOURS.

|___|___|___|
ENTER NUMBER OF MINUTES OR HOURS

REFUSED 7777 (PAQ.680)
DON'T KNOW 9999 (PAQ.680)

|___|
ENTER UNIT

MINUTES 1
HOURS 2

PAQ.680 Q/U The following question is about sitting at work, at home, getting to and from places, or with friends, including time spent sitting at a desk, traveling in a car or bus, reading, playing cards, watching television, or using a computer. Do not include time spent sleeping.

How much time {do you/does SP} usually spend sitting on a typical day?

|___|___|___|
ENTER NUMBER OF MINUTES OR HOURS

REFUSED .... 7777 (BOX 2)
DON'T KNOW .... 9999 (BOX 2)

|___|
ENTER UNIT

MINUTES 1
HOURS 2

SOFT EDIT: 18 HOURS OR MORE.
ERROR MESSAGE: PLEASE VERIFY TIMES OF 18 HOURS OR MORE.

HARD EDIT: 24 HOURS OR MORE.
ERROR MESSAGE: THE TIME SHOULD BE LESS THAN 24 HOURS.

BOX 2
**CHECK ITEM PAQ.720:**<br>IF SP AGE 18+, GO TO NEXT SECTION.

PAQ.706 Now I'd like to ask you some questions about {your/SP's} activities.

During the **past 7 days**, on how many days {were you/was SP} physically active for a total of **at least 60 minutes per day**? Add up all the time {you/he/she} spent in any kind of physical activity that increased {your/his/her} heart rate and made {you/him/her} breathe hard some of the time.

0 days	0
1 day	1
2 days	2
3 days	3
4 days	4
5 days	5
6 days	6
7 days	7
REFUSED	77
DON'T KNOW	99

PAQ.710 Now I will ask you first about TV watching and then about computer use.

Over the past 30 days, on average how many **hours per day** did {you/SP} sit and watch TV or videos? Would you say . . .

less than 1 hour,	0
1 hour,	1
2 hours,	2
3 hours,	3
4 hours,	4
5 hours or more, or	5
{You do/SP does} not watch TV or videos	8
REFUSED	77
DON'T KNOW	99

PAQ.715 Over the past 30 days, on average how many **hours per day** did {you/SP} use a computer or play computer games outside of school? Include time spent on things such as Xbox, PlayStation, an iPod, an iPad or other

tablet, a smart phone, YouTube, Facebook or other social networking tools, and the internet. Would you say . . .

less than 1 hour,	0
1 hour,	1
2 hours,	2
3 hours,	3
4 hours,	4
5 hours or more, or	5
{You do/SP does} not use a computer outside of work or school	8
REFUSED	77
DON'T KNOW	99

HELP SCREEN:
If the SP watches T.V. or video at the same time as working on the computer, count this time as watching T.V. or video.

SMOKING AND TOBACCO USE – SMQ
Target Group: SPs 0-11 years and 18+

**BOX 0**
**CHECK ITEM SMQ.005:**<br>IF SP >= 18 YEARS, CONTINUE.<br>IF SP 12-17 YEARS, GO TO END OF SECTION.<br>ELSE GO TO BOX 5.

These next questions are about cigarette smoking. Then I will ask about other tobacco products.

SMQ.022 {Have you/Has SP} smoked at least 100 **cigarettes** in {your/his/her} entire life? This hand card shows you the products we would like you to include and not include when answering this question.

HAND CARD SMQ1

YES 1
NO 2 (SMQ.890)
REFUSED 7 (SMQ.890)
DON'T KNOW 9 (SMQ.890)

SMQ.030 G/Q How old {were you/was SP} when {you/s/he} first started to smoke cigarettes regularly?

ENTER AGE 1
NEVER SMOKED CIGARETTES REGULARLY 2 (SMQ.040)
REFUSED 7 (SMQ.040)
DON'T KNOW 9 (SMQ.040)

CAPI INSTRUCTION:
SOFT EDIT: SP AGE <13
DISPLAY "UNLIKELY RESPONSE. PLEASE VERIFY."

|___|___|___|
ENTER AGE IN YEARS

REFUSED 77777
DON'T KNOW 99999

HELP SCREEN:
"regularly" refers to age when started smoking cigarettes on a routine basis as opposed to age when tried first cigarette.

SMQ.040 {Do you/Does SP} **now** smoke cigarettes . . .

every day, ... 1 (SMQ.078)
some days, or 2 (SMQ.641)
not at all? 3
REFUSED 7 (SMQ.890)
DON'T KNOW 9 (SMQ.890)

SMQ.050 Q/U How long has it been since {you/SP} quit smoking cigarettes?

|___|___|___|
ENTER NUMBER (OF DAYS, WEEKS, MONTHS OR YEARS)

REFUSED 77777
DON'T KNOW 99999

|___|
ENTER UNIT

DAYS... 1
WEEKS 2
MONTHS... 3
YEARS 4

BOX 1A

OMITTED

SMQ.057 At that time, about how many cigarettes did {you/SP} **usually** smoke a day?

1 PACK EQUALS 20 CIGARETTES
IF LESS THAN 1 PER DAY, ENTER 1
IF 95 OR MORE PER DAY, ENTER 95

|___|___|___|
ENTER NUMBER OF CIGARETTES (PER DAY)

REFUSED 7777
DON'T KNOW 9999

BOX 1B

CHECK ITEM SMQ.060:
GO TO SMQ.890.

SMQ.078 How soon after {you/SP} wake{s} up {do you/does s/he} smoke? Would you say . . .

within 5 minutes,... 1
from 6 to 30 minutes,.................................... 2
from more than 30 minutes to 1 hour,............ 3
from more than 1 hour to 2 hours, ... 4
from more than 2 hours to 3 hours, ... 5
from more than 3 hours to 4 hours, or 6
more than 4 hours? 7
REFUSED 77
DON'T KNOW 99

SMQ.641 On how many of the past **30 days** did {you/SP} smoke cigarettes?

|___|___|
ENTER NUMBER OF DAYS

REFUSED 7777
DON'T KNOW 9999

CAPI INSTRUCTION:
ALLOW '0' AS AN ENTRY. IF '0' DK OR RF ENTERED, SKIP TO QUESTION SMQ.093.

SMQ.650 On average, when {you/SP} smoked during the past **30 days**, how many cigarettes did {you/s/he} smoke a day?

1 PACK EQUALS 20 CIGARETTES
IF LESS THAN 1 PER DAY, ENTER 1
IF 95 OR MORE PER DAY, ENTER 95

|___|___|___|
ENTER NUMBER OF CIGARETTES (PER DAY)

REFUSED 7777
DON'T KNOW 9999

SMQ.093 May I please see the pack for the brand of cigarettes {you **usually** smoke/SP **usually** smokes}.

TO OBTAIN ACCURATE PRODUCT INFORMATION, IT IS IMPORTANT THAT YOU SEE THE CIGARETTE PACK.

PACK SEEN... 1
PACK NOT SEEN 2 (SMQ.100k)
NO USUAL BRAND...................................... 3 (SMQ.670)
ROLLS OWN CIGARETTES 4 (SMQ.670)
REFUSED 7 (SMQ.100k)

SMQ.310 ENTER THE UNIVERSAL PRODUCT CODE FROM THE BARCODE ON THE CIGARETTE PACK. UPC MUST CONTAIN **8 OR 12** DIGITS.

SELECT ONE OPTION.

ENTERING 8 DIGIT UPC............................. 1
ENTERING 12 DIGIT UPC........................... 2 (SMQ.330)
UNABLE TO READ CODE-PACK DAMAGED 3 (SMQ.100k)

SMQ.320 ENTER THE 8 DIGIT UPC CODE.

|___|___|___|___|___|___|___|___|

CAPI INSTRUCTION:

DOUBLE ENTRY IS REQUIRED. IF ENTRIES DO NOT MATCH, DISPLAY THE FOLLOWING MESSAGE: ENTRIES DO NOT MATCH. HIGHLIGHT THE ENTRY THAT SHOULD BE CORRECTED AND PRESS 'ENTER' TO CHANGE.

**BOX 2B**
**CHECK ITEM SMQ.329:**<br>GO TO BOX 3.

SMQ.330 ENTER THE 12 DIGIT UPC CODE.

|___|___|___|___|___|___|___|___|___|___|___|___|

CAPI INSTRUCTION:

DOUBLE ENTRY IS REQUIRED. IF ENTRIES DO NOT MATCH, DISPLAY THE FOLLOWING MESSAGE: ENTRIES DO NOT MATCH. HIGHLIGHT THE ENTRY THAT SHOULD BE CORRECTED AND PRESS 'ENTER' TO CHANGE.

**BOX 3**
**CHECK ITEM SMQ.096A:**<br>IF INVALID CODE OR CODE NOT ON FILE, GO TO SMQ.099.<br>OTHERWISE, CONTINUE.

SMQ.098 YOU HAVE SELECTED

{DISPLAY BRAND ASSOCIATED WITH CODE}

CORRECT	1	(SMQ.670)
NOT CORRECT	2	(SMQ.100k)

CAPI INSTRUCTION:
DISPLAY BRAND NAME WITH ALL QUALIFIERS – NAME, SIZE/LENGTH (REGULAR, KING, LONG, ULTRA LONG), FILTERED/NONFILTERED, MENTHOL/NONMENTHOL.
IF SMQ098 = 1 (CORRECT) AND SMQ310 = 1 (ENTER 8 DIGIT UPC), PREFILL THE FOLLOWING::

SMQ094A = UPC8 CODE
SMQ094B = UPC8 PRODUCT DESCRIPTION
SMQ100 = UPC8 CIGARETTE BRAND
SMQ110A = UPC8 FILTER
SMQ110B = UPC8 MENTHOL
SMQ110F = UPC8 SIZE/LENGTH

IF SMQ098 = 1 (CORRECT) AND SMQ310 = 2 (ENTER 12 DIGIT UPC), PREFILL THE FOLLOWING:

SMQ094A = UPC12 CODE
SMQ094B = UPC12 PRODUCT DESCRIPTION
SMQ100 = UPC12 CIGARETTE BRAND
SMQ110A = UPC12 FILTER
SMQ110B = UPC12 MENTHOL
SMQ110F = UPC12 SIZE AND LENGTH

SMQ.099 CODE NOT ON FILE – PRESS ‘ENTER’ TO CONTINUE

SMQ.100k What brand of cigarettes {do you/does SP} **usually** smoke? This hand card has pictures with names of many popular brands.

HAND CARD SMQ5

CAPI INSTRUCTION:
FOLLOW THE BASIC FORMAT FOR DIETARY SUPPLEMENT LOOKUP. ONLY ALLOW INTERVIEWER TO ENTER 1 BRAND OF CIGARETTES.

INTERVIEWER INSTRUCTION: REGULAR (68-72 MM), KING (79-88 MM), LONG (94-101 MM), ULTRA LONG (110-121 MM).

REFER TO PRODUCT LABEL IF AVAILABLE.

ENTER **BRAND** NAME OF CIGARETTE.

SMQ.111 PRESS BS TO START THE LOOKUP.

SELECT PRODUCT FROM
LIST OR TYPE NAME IDENTIFIED FROM THE HAND CARD

INTERVIEWER INSTRUCTION: REGULAR (68-72 MM), KING (79-88 MM), LONG (94-101 MM), ULTRA LONG (110-121 MM).

IF PRODUCT **NOT** ON LIST.
PRESS BS TO
DELETE ENTRY.

TYPE '**'.

PRESS ENTER TO SELECT.

CAPI INSTRUCTION:
DISPLAY CAPI CIGARETTE PRODUCT LIST. INTERVIEWER SHOULD BE ABLE TO SELECT ONE PRODUCT NAME FROM LIST. IN ADDITION, INTERVIEWER SHOULD BE ABLE TO ACCEPT THE PRODUCT NAME AS IT WAS KEYED IN SMQ.100K BY TYPING IN '**'.

BOX 4A

CHECK ITEM SMQ.112:
IF '** PRODUCT NOT ON LIST' SELECTED AT SMQ.111, CONTINUE.
OTHERWISE, GO TO SMQ.670.

SMQ.110a ASK IF NECESSARY:

IS THE CIGARETTE PRODUCT FILTERED OR NON-FILTERED?

ENTER '1' FOR **FILTERED**
ENTER '0' FOR **NON-FILTERED**

CAPI INSTRUCTION:
'1' AND '0' SHOULD BE THE ONLY CODES ACCEPTED BY CAPI.

FILTERED	1
NON-FILTERED	0
REFUSED	7777
DON'T KNOW	9999

SMQ.110b ASK IF NECESSARY:

IS THE CIGARETTE PRODUCT MENTHOL OR NON-MENTHOL?

ENTER '1' FOR **MENTHOL**
ENTER '0' FOR **NON-MENTHOL**

CAPI INSTRUCTION:
'1' AND '0' SHOULD BE THE ONLY CODES ACCEPTED BY CAPI.

MENTHOL	1
NON-MENTHOL	0
REFUSED	7777
DON'T KNOW	9999

SMQ.110h ASK IF NECESSARY:

WHAT IS THE CIGARETTE PRODUCT SIZE?

INTERVIEWER INSTRUCTION: "KINGS" ARE THE MOST POPULAR SIZE AND NOT USUALLY SHOWN ON THE PACK.

CAPI INSTRUCTION:
THIS ITEM IS STORED IN SMQ.110f IN THE DATA BASE.

REGULARS (68-72 MM)	1
KINGS (79-88 MM)	2
LONGS (94-101 MM)	3
ULTRA LONGS (110-121 MM)	4
REFUSED	7777
DON'T KNOW	9999

SMQ.670 During the past **12 months,** {have you/has SP} stopped smoking for one day or longer **because {you were/he was/she was} trying to quit smoking?**

YES	1	
NO	2	(SMQ.890)
REFUSED	7	(SMQ.890)
DON'T KNOW	9	(SMQ.890)

SMQ.848 During the past 12 months, how many times {have you/has SP} stopped smoking cigarettes because {you were/he was/she was} trying to quit smoking?

|___|___|
ENTER NUMBER OF TIMES (1-20 TIMES)

REFUSED	777
DON'T KNOW	999

CAPI INSTRUCTION:
IF MORE THAN 20 TIMES ENTER 20

SMQ.852 Q/U

The last time {you/SP} tried to quit, how long {were you/was he/was she} able to stop smoking?

CAPI INSTRUCTION:
SOFT EDIT: SMQ.852 CANNOT BE GREATER THAN 364 DAYS, 11 MONTHS OR 51 WEEKS.

|___|___|___|
ENTER NUMBER (OF DAYS, WEEKS OR MONTHS)

REFUSED 7777
DON'T KNOW 9999

ENTER UNIT

DAYS.. 1
WEEKS 2
MONTHS.. 3
REFUSED 7
DON'T KNOW 9

BOX 4B

OMITTED

SMQ.890 {Have you/Has SP} **ever** smoked a regular cigar, cigarillo or little filtered cigar **even one time**? This hand card shows examples of some cigars; however there are others not included here.

HAND CARD SMQ2

YES 1
NO 2 (SMQ.900)
REFUSED 7 (SMQ.900)
DON'T KNOW 9 (SMQ.900)

HELP TEXT: A cigar is defined, for tax purposes, as: Any roll of tobacco wrapped in leaf tobacco or in any substance containing tobacco.

SMQ.895 During the past **30 days**, on how many days did {you/SP} smoke a regular cigar, cigarillo or little filtered cigar?

|___|___|
ENTER NUMBER OF DAYS

REFUSED 77
DON'T KNOW 99

CAPI INSTRUCTION: ALLOW '0' AS AN ENTRY.

SMQ.900 The next question is about e-cigarettes. These are battery-powered devices that usually contain liquid nicotine, and don't produce smoke

{Have you/Has SP} **ever** used an e-cigarette **even one time**? This hand card shows examples of some e-cigarettes and other devices used to inhale liquid nicotine; however there are others not included here.

HAND CARD SMQ3

INTERVIEWER: USE OF THESE DEVICES FOR MARIJUANA OR SUBSTANCES OTHER THAN NICOTINE SHOULD NOT BE COUNTED.

YES 1
NO 2 (SMQ.910)
REFUSED 7 (SMQ.910)
DON'T KNOW 9 (SMQ.910)

HELP SCREEN for SMQ.900: E-cigarettes, e-hookahs, vape pens and other similar products are bought as disposable or reusable kits with a cartridge or with refillable container. They contain nicotine or flavored liquid, called "e-liquid" or "e-juice".

SMQ.905 During the past **30 days**, on how many days did {you/SP} use e-cigarettes?

|___|___|
ENTER NUMBER OF DAYS

REFUSED 77
DON'T KNOW 99

CAPI INSTRUCTION: ALLOW '0' AS AN ENTRY.

SMQ.910 Smokeless tobacco products are placed in the mouth and nose and include chewing tobacco, snuff, dip, snus (pronounced as "snoose") and dissolvable tobacco.

{Have you/Has SP} **ever** used smokeless tobacco **even one time**? This hand card shows examples of smokeless products; however there are others not included here.

HAND CARD SMQ4

YES 1
NO 2 (SMQ.856)
REFUSED 7 (SMQ.856)
DON'T KNOW 9 (SMQ.856)

SMQ.915 During the past **30 days**, on how many days did {you/SP} use smokeless tobacco?

|___|___|
ENTER NUMBER OF DAYS

REFUSED 77
DON'T KNOW 99

CAPI INSTRUCTION: ALLOW '0' AS AN ENTRY.

BOX 5
**CHECK ITEM SMQ.854:**<br>IF SP AGE 0-11, GO SMQ.860.<br>OTHERWISE, CONTINUE.

SMQ.856 I will now ask you about tobacco smoke in other places.

During the last 7 days, {were you/was SP} working at **a job or business outside of the home?**

YES	1	
NO	2	(SMQ.860)
REFUSED	7	(SMQ.860)
DON'T KNOW	9	(SMQ.860)

SMQ.858 While {you were/SP was} **working at a job or business outside of the home,** did someone else smoke cigarettes or other tobacco products indoors?

YES	1
NO	2
REFUSED	7
DON'T KNOW	9

SMQ.860 {I will now ask you about smoking in other places.} During the last 7 days, did {you/SP} spend time in a **restaurant?**

YES	1	
NO	2	(BOX 6)
REFUSED	7	(BOX 6)
DON'T KNOW	9	(BOX 6)

CAPI INSTRUCTION:
DISPLAY ‘I will now ask you about smoking in other places’ IF SP AGE 0-11 YEARS.

SMQ.862 While {you were/SP was} in a **restaurant**, did someone else smoke cigarettes or other tobacco products indoors?

YES	1
NO	2
REFUSED	7
DON'T KNOW	9

BOX 6
**CHECK ITEM SMQ.864:**<br>IF SP >=18 YEARS, CONTINUE.<br>OTHERWISE, GO TO SMQ.870.

SMQ.866 During the last 7 days, {did you/SP} spend time in **a bar?**

YES	1	
NO	2	(SMQ.870)
REFUSED	7	(SMQ.870)
DON'T KNOW	9	(SMQ.870)

SMQ.868 While {you were/SP was} in **a bar,** did someone else smoke cigarettes or other tobacco products indoors?

YES	1
NO	2
REFUSED	7
DON'T KNOW	9

SMQ.870 During the last 7 days, did {you/SP} ride in a **car or motor vehicle?**

YES	1	
NO	2	(SMQ.874)
REFUSED	7	(SMQ.874)
DON'T KNOW	9	(SMQ.874)

SMQ.872 While {you were/SP was} riding in **a car or motor vehicle,** did someone else smoke cigarettes or other tobacco products?

YES	1
NO	2
REFUSED	7
DON'T KNOW	9

SMQ.874 During the last 7 days, did {you/SP} spend time in **a home other than {your/his/her} own?**

YES	1	
NO	2	(SMQ.878)
REFUSED	7	(SMQ.878)
DON'T KNOW	9	(SMQ.878)

SMQ.876 While {you were/SP was} in **a home other than {your/his/her} own,** did someone else smoke cigarettes or other tobacco products indoors?

YES	1
NO	2
REFUSED	7
DON'T KNOW	9

SMQ.878 During the last 7 days,{were you/was SP} in **any other indoor area?**

INTERVIEWER: IF RESPONDENT ASKS WHAT IS MEANT BY OR DOESN'T SEEM TO UNDERSTAND "ANY OTHER INDOOR AREA" SAY "OTHER THAN AT WORK, IN A BAR, RESTAURANT, CAR, OTHER MOTOR VEHICLE, OR A HOUSE."

YES	1	
NO	2	(SMQ.940)
REFUSED	7	(SMQ.940)
DON'T KNOW	9	(SMQ.940)

SMQ.880 While {you were/SP was} in the **other indoor** area, did someone else smoke cigarettes or other tobacco products?

YES	1
NO	2
REFUSED	7
DON'T KNOW	9

SMQ.940 The next question is about e-cigarettes.

During the last 7 days, {were you/was SP} in an **indoor** place where someone was using an e-cigarette, e-hookah, vape-pen or other similar electronic product?

YES	1
NO	2
REFUSED	7
DON'T KNOW	9

SLEEP DISORDERS – SLQ
Target Group: 16+

SLQ.300 The next set of questions is about {your/SP's} sleep and work behavior.

What time {do you/does SP} usually fall asleep on weekdays or workdays?

|__|__| : |__|__| ENTER AM OR PM
HH MM

INTERVIEWER INSTRUCTION: THIS IS NOT THE TIME SP GETS INTO BED. ENTER TIME AS HH:MM AM OR PM. IF RESPONDENT SAYS TWELVE "MIDNIGHT" CODE AS 12:00 **AM**.

REFUSED 77777777
DON'T KNOW 99999999

SLQ.310 What time {do you/does SP} usually wake up on weekdays or workdays?

|__|__| : |__|__| ENTER AM OR PM
HH MM

INTERVIEWER INSTRUCTION: THIS IS NOT THE TIME SP GETS OUT OF BED. ENTER TIME AS HH:MM AM OR PM.

REFUSED 77777777
DON'T KNOW 99999999

CAPI INSTRUCTION:
SOFT EDIT: LESS THAN 4 HOURS OR MORE THAN 12 HOURS OF TOTAL SLEEP. IF SLQ.300 OR 310 IS DK OR RF, DO NOT APPLY SOFT EDIT.
ERROR MESSAGE: PLEASE VERIFY SLEEP TIMES OF LESS THAN 4 HOURS OR MORE THAN 12 HOURS.

SLQ.320 What time {do you/does SP} usually fall asleep on weekends or non-workdays?

|__|__| : |__|__| ENTER AM OR PM
HH MM

INTERVIEWER INSTRUCTION: THIS IS NOT THE TIME SP GETS INTO BED.
INTERVIEWER INSTRUCTION: ENTER TIME AS HH:MM AM OR PM. IF RESPONDENT SAYS TWELVE "MIDNIGHT" CODE AS 12:00 **AM**.

INTERVIEWER INSTRUCTION: IF RESPONDENT SAYS DOES NOT WORK, ASK IF THE TIME THAT THE RESPONDENT FALLS ASLEEP IS DIFFERENT ON WEEKENDS. IF NOT, ENTER SAME TIME AS SLQ.300.

REFUSED 77777777
DON'T KNOW 99999999

SLQ.330 What time {do you/does SP} usually wake up on weekends or non-workdays?

|__|__| : |__|__| ENTER AM OR PM
HH MM

INTERVIEWER INSTRUCTION: THIS IS NOT THE TIME SP GETS OUT OF BED.
INTERVIEWER INSTRUCTION: ENTER TIME AS HH:MM AM OR PM.

REFUSED 77777777
DON'T KNOW 99999999

INTERVIEWER INSTRUCTION: IF RESPONDENT SAYS DOES NOT WORK, ASK IF THE TIME THAT THE RESPONDENT WAKES UP IS DIFFERENT ON WEEKENDS. IF NOT, ENTER SAME TIME AS SLQ.310.

CAPI INSTRUCTION:
SOFT EDIT: LESS THAN 4 HOURS OR MORE THAN 12 HOURS OF TOTAL SLEEP. IF SLQ.320 OR 330 IS DK OR RF, DO NOT APPLY SOFT EDIT.
ERROR MESSAGE: PLEASE VERIFY SLEEP TIMES OF LESS THAN 4 HOURS OR MORE THAN 12 HOURS.

SLQ.030 **In the past 12 months,** how often did {you/SP} snore while {you were/s/he was} sleeping?

INTERVIEWER INSTRUCTION: IF R SAYS “DON’T KNOW”, PROBE IF ANYONE HAS TOLD THEM THAT THEY SNORE.

Never, ... 0
Rarely – 1-2 nights a week, ... 1
Occasionally – 3-4 nights a week, or 2
Frequently – 5 or more nights a week? 3
REFUSED 7
DON’T KNOW 9

SLQ.040 **In the past 12 months,** how often did {you/SP} snort, gasp, or stop breathing while {you were/s/he was} asleep?

INTERVIEWER INSTRUCTION: IF THE RESPONDENT ASKS “HOW WOULD I KNOW IF I SNORT, GASP OR STOP BREATHING WHEN I AM SLEEPING? PROBE IF ANYONE TOLD THEM THAT THEY DO THIS.

Never, ... 0
Rarely – 1-2 nights a week, ... 1
Occasionally – 3-4 nights a week, or 2
Frequently – 5 or more nights a week? 3
REFUSED 7
DON’T KNOW 9

SLQ.050 {Have **you**/Has SP} **ever told** a doctor or other health professional that {you have/s/he has} trouble sleeping?

YES 1
NO 2
REFUSED 7
DON'T KNOW 9

SLQ.120 **In the past month,** how often did {you/SP} feel excessively or overly sleepy during the day?

HAND CARD SLQ1

NEVER	0
RARELY – 1 TIME A MONTH	1
SOMETIMES – 2-4 TIMES A MONTH	2
OFTEN – 5-15 TIMES A MONTH	3
ALMOST ALWAYS – 16-30 TIMES A MONTH	4
REFUSED	7
DON'T KNOW	9
